# 757 Focusing the Lens on Burn Wound Photography: An Institutional Assessment

**DOI:** 10.1093/jbcr/irae036.299

**Published:** 2024-04-17

**Authors:** Nicolas Malkoff, Brigette Cannata, Artur Manasyan, Idean Roohani, Tayla Moshal, Elaine Terr, Misato Koizumi, Maxwell B Johnson, Justin Gillenwater

**Affiliations:** Keck School of Medicine of USC, Carlsbad, CA; Keck School of Medicine of USC, Staten Island, NY; Keck School of Medicine, University of Southern California, Los Angeles, CA; Keck School of Medicine of USC, Los Angeles, CA; Keck School of Medicine, Los Angeles, CA; Los Angeles General Medical Center (GEN), altadena, CA; Los Angeles General Hospital, Woodland Hills, CA; Los Angeles General Medical Center, Arcadia, CA; Los Angeles General Medical Regional Burn Center, Los Angeles, CA; University of Southern California, Los Angeles, CA; Keck Medicine of USC, Los Angeles, CA; Keck School of Medicine of USC, Carlsbad, CA; Keck School of Medicine of USC, Staten Island, NY; Keck School of Medicine, University of Southern California, Los Angeles, CA; Keck School of Medicine of USC, Los Angeles, CA; Keck School of Medicine, Los Angeles, CA; Los Angeles General Medical Center (GEN), altadena, CA; Los Angeles General Hospital, Woodland Hills, CA; Los Angeles General Medical Center, Arcadia, CA; Los Angeles General Medical Regional Burn Center, Los Angeles, CA; University of Southern California, Los Angeles, CA; Keck Medicine of USC, Los Angeles, CA; Keck School of Medicine of USC, Carlsbad, CA; Keck School of Medicine of USC, Staten Island, NY; Keck School of Medicine, University of Southern California, Los Angeles, CA; Keck School of Medicine of USC, Los Angeles, CA; Keck School of Medicine, Los Angeles, CA; Los Angeles General Medical Center (GEN), altadena, CA; Los Angeles General Hospital, Woodland Hills, CA; Los Angeles General Medical Center, Arcadia, CA; Los Angeles General Medical Regional Burn Center, Los Angeles, CA; University of Southern California, Los Angeles, CA; Keck Medicine of USC, Los Angeles, CA; Keck School of Medicine of USC, Carlsbad, CA; Keck School of Medicine of USC, Staten Island, NY; Keck School of Medicine, University of Southern California, Los Angeles, CA; Keck School of Medicine of USC, Los Angeles, CA; Keck School of Medicine, Los Angeles, CA; Los Angeles General Medical Center (GEN), altadena, CA; Los Angeles General Hospital, Woodland Hills, CA; Los Angeles General Medical Center, Arcadia, CA; Los Angeles General Medical Regional Burn Center, Los Angeles, CA; University of Southern California, Los Angeles, CA; Keck Medicine of USC, Los Angeles, CA; Keck School of Medicine of USC, Carlsbad, CA; Keck School of Medicine of USC, Staten Island, NY; Keck School of Medicine, University of Southern California, Los Angeles, CA; Keck School of Medicine of USC, Los Angeles, CA; Keck School of Medicine, Los Angeles, CA; Los Angeles General Medical Center (GEN), altadena, CA; Los Angeles General Hospital, Woodland Hills, CA; Los Angeles General Medical Center, Arcadia, CA; Los Angeles General Medical Regional Burn Center, Los Angeles, CA; University of Southern California, Los Angeles, CA; Keck Medicine of USC, Los Angeles, CA; Keck School of Medicine of USC, Carlsbad, CA; Keck School of Medicine of USC, Staten Island, NY; Keck School of Medicine, University of Southern California, Los Angeles, CA; Keck School of Medicine of USC, Los Angeles, CA; Keck School of Medicine, Los Angeles, CA; Los Angeles General Medical Center (GEN), altadena, CA; Los Angeles General Hospital, Woodland Hills, CA; Los Angeles General Medical Center, Arcadia, CA; Los Angeles General Medical Regional Burn Center, Los Angeles, CA; University of Southern California, Los Angeles, CA; Keck Medicine of USC, Los Angeles, CA; Keck School of Medicine of USC, Carlsbad, CA; Keck School of Medicine of USC, Staten Island, NY; Keck School of Medicine, University of Southern California, Los Angeles, CA; Keck School of Medicine of USC, Los Angeles, CA; Keck School of Medicine, Los Angeles, CA; Los Angeles General Medical Center (GEN), altadena, CA; Los Angeles General Hospital, Woodland Hills, CA; Los Angeles General Medical Center, Arcadia, CA; Los Angeles General Medical Regional Burn Center, Los Angeles, CA; University of Southern California, Los Angeles, CA; Keck Medicine of USC, Los Angeles, CA; Keck School of Medicine of USC, Carlsbad, CA; Keck School of Medicine of USC, Staten Island, NY; Keck School of Medicine, University of Southern California, Los Angeles, CA; Keck School of Medicine of USC, Los Angeles, CA; Keck School of Medicine, Los Angeles, CA; Los Angeles General Medical Center (GEN), altadena, CA; Los Angeles General Hospital, Woodland Hills, CA; Los Angeles General Medical Center, Arcadia, CA; Los Angeles General Medical Regional Burn Center, Los Angeles, CA; University of Southern California, Los Angeles, CA; Keck Medicine of USC, Los Angeles, CA; Keck School of Medicine of USC, Carlsbad, CA; Keck School of Medicine of USC, Staten Island, NY; Keck School of Medicine, University of Southern California, Los Angeles, CA; Keck School of Medicine of USC, Los Angeles, CA; Keck School of Medicine, Los Angeles, CA; Los Angeles General Medical Center (GEN), altadena, CA; Los Angeles General Hospital, Woodland Hills, CA; Los Angeles General Medical Center, Arcadia, CA; Los Angeles General Medical Regional Burn Center, Los Angeles, CA; University of Southern California, Los Angeles, CA; Keck Medicine of USC, Los Angeles, CA

## Abstract

**Introduction:**

Wound photography plays a critical role in burn care. In addition to written descriptions, photographs document wound changes and inform management decisions. Furthermore, photographs may be utilized for academic purposes such as teaching and research. With the development of smartphones, clinical photography has become faster and easier but has seen a decline in consistency and overall image quality. This study aims to evaluate the quality of burn wound photography at our institution to identify areas for improvement and inform efforts to develop a standardized photography protocol.

**Methods:**

A wound photography rating scale with seven categories and 14 total points was developed based on published recommendations. Individual categories include background (2 max), distractors (2 max), lighting (2 max), camera position (1 max), wound capture (3 max), anatomic orientation (2 max), and patient ID and scale (2 max). Total scores are classified as follows: 0-4 “poor,” 5-9 “fair,” and 10-14 “good.” Camera focus was not included, as blurred photographs are removed by a medical photographer prior to entry into patient records at our institution. Admission wound photographs of 100 randomly sampled patients admitted to the burn unit of a large, urban medical center from 1/1/22 to 8/31/23 were extracted from the medical record for evaluation. Each photograph was independently evaluated by three reviewers, and final scores were determined using a Delphi consensus method. Results are reported using descriptive statistics.

**Results:**

Of the 100 patients included for review, 12 were missing admission photographs of their burn wounds. The 88 photographs that were evaluated received a mean score of 7.7 ± 2.3 out of 14 on the burn wound photography rating scale. Only 17% of photographs received a score of 10 or more, and 7% received a score of 4 or less. A solid-colored or white backdrop was present in 2% and 39% of photographs, respectively. Only 9% of photographs were free of unnecessary distractors. Ideal lighting without shadowing or washout was used in 36% of photographs. The camera lens was positioned parallel to the wound in 80% of photographs. The entirety of the wound bed and periwound skin was captured in 67% of the photographs. In two photographs (2%), the reviewers were unable to determine anatomic orientation. Patient ID was included in 98% of photographs while a scale, such as a ruler, was included in only one photograph.

**Conclusions:**

Current burn wound photography practices at our institution are inconsistent and fail to meet quality standards. The data collected in this study will be used to develop a standardized wound photography and documentation algorithm for integration into the existing burn unit workflow.

**Applicability of Research to Practice:**

Readers may consider the methodology and findings of this study in evaluating the wound photography practices at their own institutions.

